# PB.23. Breast density in previous screening mammograms of women with and without breast cancer

**DOI:** 10.1186/bcr3717

**Published:** 2014-11-03

**Authors:** L Sivagnanam, A Hufton, M Berks, E Harkness, Y Lim, A Maxwell, M Wilson, M Bydder, S Gadde, DG Evans, A Howell, P Stavrinos, S Astley

**Affiliations:** 1 University of Manchester Medical School, Manchester, UK; 2 Centre for Imaging Sciences, Institute of Population Health, University of Manchester, UK; 3 Nightingale Centre and Genesis Prevention Centre, University Hospital of South Manchester, Manchester, UK; 4 Manchester Breast Centre, Manchester Cancer Research Centre, University of Manchester, Christie Hospital, Manchester, UK; 5 The University of Manchester, Manchester Academic Health Science Centre, University Hospital of South Manchester NHS Foundation Trust, Manchester, UK

## Introduction

Increased mammographic density is a well-established risk factor for breast cancer; much of the evidence is based on semi-automated or visual assessment of analogue mammograms, although volumetric measures from digitally acquired mammograms are now being reported. It is also possible to quantify volumes of fat and gland from digitised analogue mammograms, given suitable calibration data. We have measured breast density in cancer cases and controls which had previous analogue screening mammograms in the PROCAS (Predicting Risk Of Cancer At Screening) study.

## Methods

Forty-nine (44 screen-detected, five interval) cancer cases with film priors were each matched to one control without cancer on the basis of age, BMI, menopausal status and current HRT use. The previous normal screening mammograms for each case were digitised and volumetric breast density measured in the CC view using a calibrated step-wedge imaged alongside the breast. Average area-based percent density was obtained from two readers recording assessments on 10 cm visual analogue scales (VAS).

## Results

Median volumetric percent density was 4.74% (cancer priors) and 4.77% (controls); this was not a significant difference (*P *= 0.436). For gland volume the corresponding figures were 37.0 cm^3 ^and 32.1 cm^3 ^(*P *= 0.667), and for VAS percent density were 28.8% and 27.8% (*P *= 0.538).

## Conclusion

In this sample we did not detect a significant difference between density in prior mammograms of cancer cases and those of controls. The sample size, however, was small. Increasing availability of digital priors of screen-detected cancers will facilitate further exploration of the ability of increased density to predict the development of cancer.

